# Assessing the effect of metastasis-directed therapy in oligometastatic disease using the restricted mean survival time

**DOI:** 10.1038/s41416-024-02700-z

**Published:** 2024-05-06

**Authors:** Itamar Averbuch, Assaf Moore, Ethan B. Ludmir, Gal Markel, Tomer Meirson

**Affiliations:** 1 Davidoff Cancer Center, Rabin Medical Center – Beilinson Hospital, Petach Tikva, Israel; 2 The University of Texas MD Anderson Cancer Center, Houston, TX USA; 3 Clinical Microbiology and Immunology, Sackler Faculty of Medicine, Tel Aviv University, Tel Aviv, Israel

**Keywords:** Metastasis, Radiotherapy

## Abstract

**Background:**

Metastasis-directed therapy (MDT) with stereotactic body radiotherapy (SBRT) is emerging as an effective therapeutic option for oligometastatic disease (OMD). However, a lack of phase III data, consensus guidelines, and toxicity concerns limit its widespread use. Randomized controlled trials (RCTs) routinely report hazard ratios (HRs) and medians that lack clear clinical and robust interpretation. Restricted-mean survival time (RMST) is the duration of time a patient is expected to survive over the follow-up period, providing a robust and interpretable alternative. We analyzed the efficacy of SBRT using RMST.

**Methods:**

All registered RCTs of ablative radiotherapy in OMD in ClinicalTrials.gov through 2022 were identified. Data were reconstructed from Kaplan–Meier curves, and the HRs and RMST differences were estimated for surrogate endpoints (SEs) and overall survival (OS).

**Results:**

Six studies comprising 426 patients met the inclusion criteria. The RMST differences for SEs ranged from 4.6 months in a study by Iyengar et al. to 11.1 months in SABR-COMET. The RMST differences for OS in SABR-COMET, Gomez et al., and SINDAS studies were 12.6, 15 and 7.9 months, respectively.

**Conclusion:**

RMST demonstrates the efficacy of local treatment in OMD. Representing the expected survival time, this method effectively communicates outcomes to patients and clinicians.

## Background

The oligometastatic paradigm differentiates between patients with low- and high-volume metastatic disease. This paradigm suggests that patients with a limited number of metastases may benefit from aggressive local treatment such as resection or ablative radiation therapy [1]. While the maximal number of lesions to be considered oligometastatic has not been clearly defined and is the subject of ongoing research [2, 3], there is some consensus for three to five metastatic lesions. SABR-COMET was the first phase II randomized controlled trial (RCT) that demonstrated the potential benefit of adding MDT with stereotactic body radiation therapy (SBRT) to standard of care systemic therapy in patients with oligo-metastatic solid tumors [4, 5]. The study found that the addition of SBRT led to an improvement of 22 months in median overall survival (OS) [4, 5].

While contemporary clinical trials are limited by factors such as small sample sizes, variations in baseline characteristics, histologies and number of lesions [5], there is a growing body of evidence suggesting that local treatment can improve outcomes in the oligometastatic setting. However, concerns remain for severe radiotherapy toxicity, negatively impacting patients’ quality of life (QoL). While reports of high-grade toxicity have been limited [6], SABR COMET recorded three treatment-related deaths [4, 5]. Therefore, treatment goals should be weighed against potential adverse effects, and physicians should be able to provide patients with clear information about the benefits in order to make an informed decision. Relevant studies have reported results using hazard ratios (HR) and median OS, harboring several limitations. The HR represents the ratio of the instantaneous event rate between treatment groups assuming proportional hazards, that is, the HR is constant over the entire study period [7]. Given the incomprehensible nature of this measure, it is customary to simultaneously report the median value. However, the median can be misleading as it is seldom incalculable. It is insensitive to short- or long-term survival and may be less stable with respect to precision, owing to a comparably large standard error and consequent wide confidence intervals [8–10].

The restricted mean survival time (RMST) is an alternative way to analyze inter-group differences. The RMST provides a clinically interpretable, global summary of survival which may be more stable than the median [8, 9, 11]. Unlike the HR, the RMST does not depend on the proportional hazards assumption, i.e., hazards remain constant over time. The RMST is calculated as the area under the Kaplan–Meier curve and represents the mean event-free survival time within a specified follow-up time. This method can be easily implemented and provide physicians and patients with an intuitive interpretation of the data.

This study aimed to quantify the effect of local treatment with radiotherapy in oligometastatic disease (OMD) in currently available data using the RMST method.

## Methods

### Data extraction

A review of the literature was conducted, and prospective studies reporting local treatments’ effect in OMD were extracted. Phase II or III RCTs that examined the impact of local therapy to all disease sites in solid malignancies were included. Radiotherapy (RT), surgery, or a combination of the two were considered local treatments, however, only studies in which the main modality for local treatment was RT were eventually included and analyzed. Studies with incomplete or unpublished results were excluded. As this study used anonymized records and deidentified data sets that exist in the public domain, no ethics committee approval was required.

### Kaplan–Meier reconstruction

When applicable, Kaplan–Meier curves for progression-free survival (PFS) and OS were extracted using WebPlotDigitizer v4.3 and rebuilt using the reconstructed KM package in R v0.1.0. This approach allows for reproduction of time-to-event data at the patient level with minimal variations between reconstructed and original data [12, 13].

### Survival analysis

The Cox proportional hazards modeling was performed using the survival package in R, v3.2-7 to calculate the HR. The RMST which is the nonparametric alternative strategy of the HR that does not rely on the proportional hazards assumption, was calculated using the survRM2 package in R, v1.0-3. The RMST difference (RMST-D), representing the area bounded by two Kaplan-Meier curves, reflects the absolute mean gain or loss in survival. The RMST-D was calculated up to the earlier of the last events from each treatment arm. *P* values of the treatment effects using the conventional method and RMST were calculated using the 2-sided unstratified log-rank and RMST tests, respectively, with *P* < 0.05 indicating statistical significance.

## Results

**Fig. 1:**
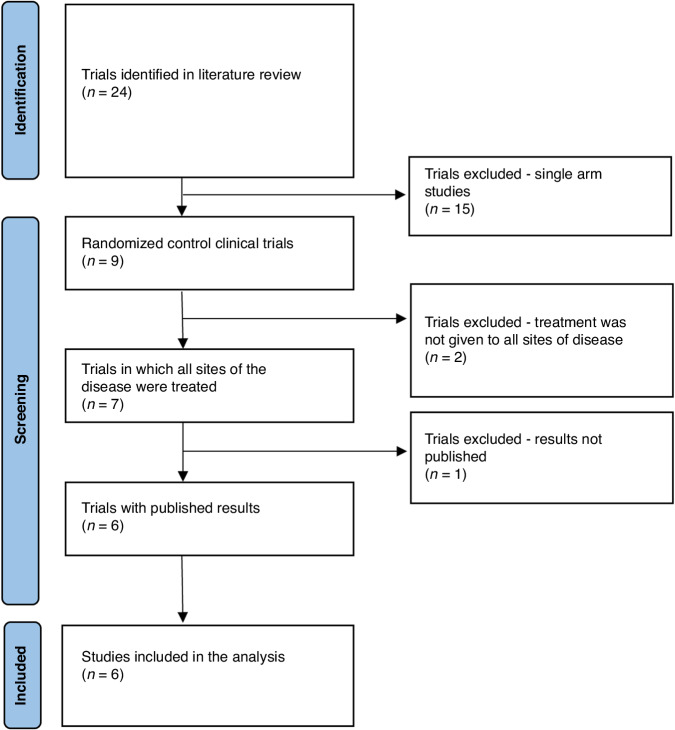
Chart flow. Diagram for the included randomized controlled trials of localized treatment for oligometastatic disease.

Twenty-four studies were examined for the inclusion criteria. Five phase II and one phase III RCTs comprising 426 patients met the inclusion criteria (Flow chart in Fig. 1, list of studies in Table 1). The SINDAS, Gomez, and SABR-COMET studies reported OS, whereas the remaining studies, reported only surrogate endpoints. PFS was reported in the Iyengar, and ORIOLE trials, and biochemical recurrence-free survival (bRFS) was reported in the STOMP and ORIOLE trials. All studies met their primary endpoint. In total, 186 patients were randomized to control groups and 240 to intervention groups. The most common primary site was lung cancer (53.8% of cases); followed by prostate cancer (31%); breast or colorectal cancer (4.2% each), and other primary malignancies (6.8%). MDT was RT alone in four RCTs and RT or surgery in two trials. In total, the MDT modality was RT, RT and surgery and surgery alone in 227 (94.6%), 6 (2.5%) and 7 (2.9%) patients, respectively. RT dose and fractionation scheme ranged between 15–24 Gray (Gy) in a single fraction, through 9.5–70 Gy in 3–12 fractions, and 45–66 Gy in 15–33 fractions. Three studies included patients with up to five metastases and three studies included patients with up to three metastases. Patients’ performance status (PS) at baseline was graded using one of the following scales: Zubrod, PS 0-2; World Health Organization (WHO), PS 0–1; Eastern Cooperative Oncology Group (ECOG), PS ≤ 2; Karnofsky, PS ≥ 70 (Additional basic characteristics are listed in Table 2).

**Table 1:** Randomized controlled trials of local ablative treatment for oligometastatic disease.

NCT No.	Name	Year	Histology	Population	Number of patients	Intervention	Synchronicity
NCT02045446	Iyengar	2018	NSCLC	ITT	29	SBRT	Sync & Meta
NCT01446744	SABR-COMET	2019	Mixed	ITT	99	SBRT	Meta
NCT01558427	STOMP	2018	Prostate	ITT	62	SBRT/Surgery	Meta
NCT02680587	ORIOLE	2020	Prostate	ITT	54	SBRT	Meta
NCT01725165	Gomez	2016	NSCLC	ITT	49	RT/ Surgery	Sync & Meta
NCT02893332	SINDAS	2022	NSCLC	ITT	133	SBRT	Sync

*NCT* National clinical trial, *NSCLC* Non-small cell lung cancer, *SBRT* stereotactic body radiation therapy, *CRT* Chemo-radiotherapy, *RT* Radiotherapy, *ITT* Intention-to-treat, *Sync* Synchronicity, *Meta* Metachronous.

**Table 2:** Baseline trial characteristics.

Characteristic (no. of trials)		Control (*n*)	Intervention (*n*)	Total (*n*)
Median age (5)^a^		68.5 [64–70]^b^	65.25 [62–68]^b^	67.5
Sex (6)	Male	115	153	268
	Female	71	87	158
Primary (6)	Lung	110	119	229
	Prostate	51	81	132
	Breast	5	13	18
	Colorectal	9	9	18
	Other	11	18	29
Synchronous (5)	Synchronous	169	225	423
	Metachronous	2	1	3
Number of metastases	1-2	91	110	201
(5)	3-4	42	53	95
	5	4	9	13

*n* number of patients.

^a^The median of the medians.

^b^Median age range.

**Fig. 2:**
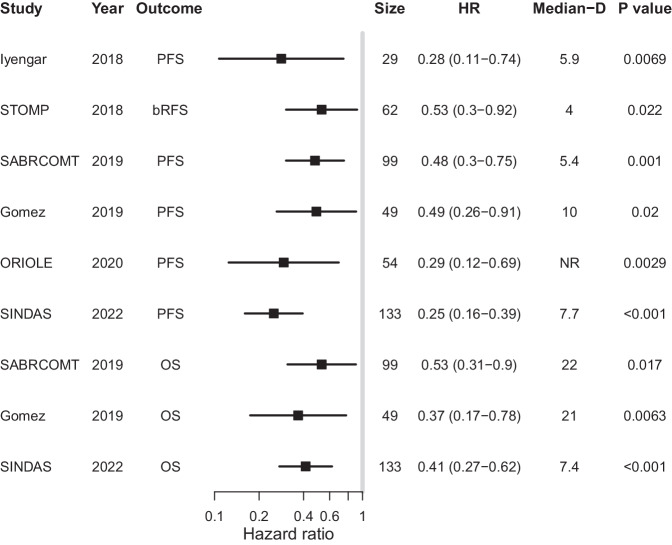
Forest plot of hazard ratios. Shown are calculated hazard ratios (HR) and difference in median (median-D) survival for surrogate endpoints and overall survival (OS). PFS progression-free survival, bRFS biochemical recurrence-free survival, Size sample size.

The calculated and reported HRs for the RCTs were highly correlated (Pearson coefficient *R* = 0.97, *P* < 0.001, Supplementary Fig. 1), indicating that the extraction and reconstruction methodology were accurate. The calculated HRs for the surrogate endpoints (SEs) and OS are shown in Fig. 2. The HRs for SEs ranged from 0.25 (95% CI 0.16–0.39, *p* < 0.001) in SINDAS to 0.53 (95% CI 0.30–0.92, *p* = 0.024) in STOMP. The HRs for OS in SABR-COMET, Gomez et al., and SINDAS were 0.48 (95% CI 0.30–0.75, *p* = 0.0013), 0.37 (95% CI 0.17–0.78, *p* = 0.0086), and 0.41 (95% CI 0.27–0.62, *p* < 0.001), respectively.

**Fig. 3:**
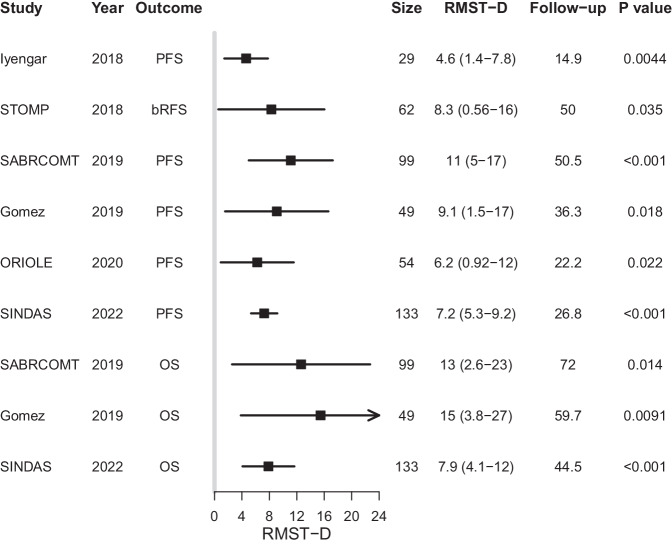
Forest plot of restricted-mean survival time difference (RMST-D). Shown are RMST-D in months for surrogate endpoints and overall survival (OS). The follow-up time indicates the earlier of the last observed individual of the two groups. PFS progression-free survival, bRFS biochemical recurrence-free survival, Size sample size.

**Fig. 4:**
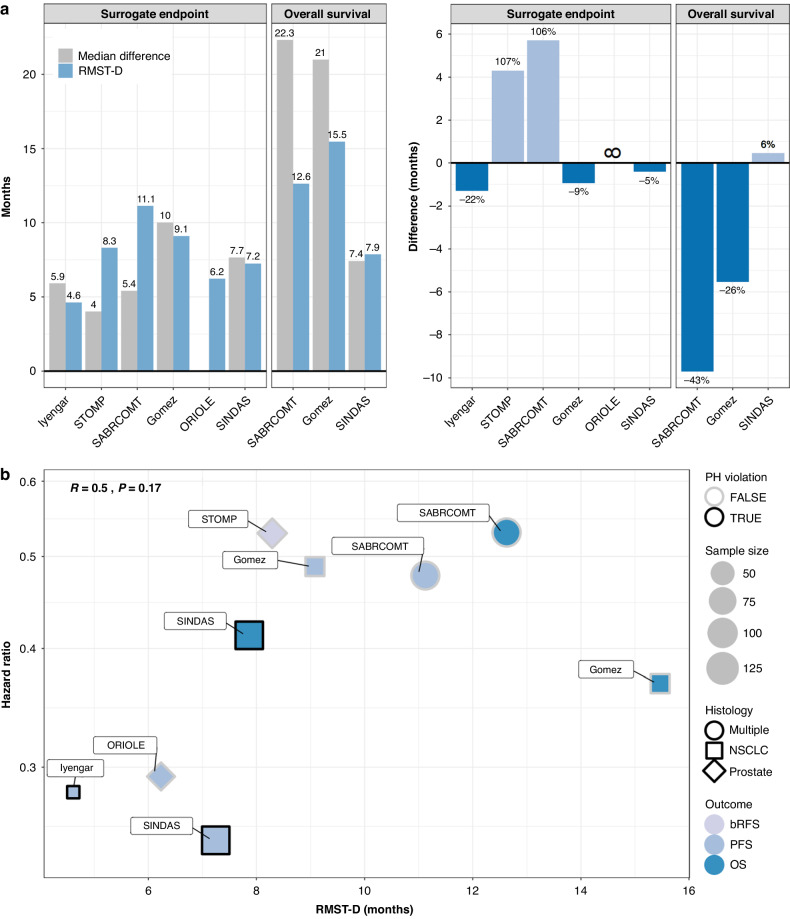
Comparison between the survival measures. **a** Shown is the comparison between the restricted-mean survival time difference (RMST-D) and the median difference in absolute (left), and relative (right) terms for the surrogate endpoints and overall survival (OS). No difference could be calculated for ORIOLE since the median was not estimable (indicated by ∞). **b** Correlation between RMST-D and hazard ratio (HR) is depicted, along with its associations with proportional hazards (PH) violation, sample size, histology, and outcome. OS overall survival, PFS progression-free survival, bRFS biochemical recurrence-free survival.

The calculated RMST-D for the SEs and OS are shown in Fig. 3. The RMST-D for SEs ranged from 4.6 months (95% CI 1.4–7.8, *p* = 0.0044) in the publication by Iyengar et al. to 11.1 months (95% CI 5.0–17.2, *p* < 0.001) in SABR-COMET. The RMST-D for OS in SABR-COMET, Gomez et al., and SINDAS were 12.6 months (95% CI 2.6–22.7, *p* = 0.014), 15 months (95% CI 3.8–27, *p* = 0.0091), and 7.9 months (95% CI 4.1–11.6, *p* < 0.001), respectively. A comparison between the RMST-D and the median differences is shown in Fig. 4a. The correlation between the RMST and HR and the association with the sample size, histology, PH violation, and outcome is shown in Fig. 4b. We found no significant correlation between the RMST-D and log_2_(HR) for all outcomes (Pearson coefficient *R* = 0.50, *P* = 0.17) or for SEs (Pearson coefficient *R* = 0.75, *P* = 0.086).

## Discussion

Based on recently published phase II/III studies, we found that individuals with OMD receiving aggressive local treatment survived an excess of 7.9–15 months and survived without disease progression, between 4.6 and 11.1 months, on average.

In the setting of OMD, SBRT as means of MDT has been endorsed as a standard-of-care treatment [14, 15]. Its main indications include residual disease after systemic treatment, cases unsuitable for surgery, or oligo-progressive disease [16, 17]. Historically, most data supporting this concept was based on single-arm studies [6]. A small number of RCTs have been conducted to examine this hypothesis. ORIOLE, and STOMP were phase II trials evaluating the addition of SBRT to all disease sites in oligometastatic prostate cancer. ORIOLE demonstrated an improved median PFS (mPFS) (not reached vs. 5.8 months, *p* = 0.002), and STOMP showed an improved median bRFS (10 vs. 6 months, *p* = 0.03), in favor of the intervention groups [4, 18, 19]. Three trials examined the effect of MDT in non-small cell lung cancer (NSCLC) patients. Gomez et al. and Iyengar et al. investigated the role of local consolidative RT in patients without progression after front-line systemic treatment. Both trials were terminated early due to significantly prolonged mPFS in the treatment arm (Gomez et al.: 14.2 vs. 4.4 months, *p* = 0.022; Iyengar et al.: 9.7 vs. 3.5 months, *p* = 0.01) [20, 21]. The sample size of SINDAS was the largest, with 133 NSCLC patients receiving first-line tyrosine kinase inhibitors. The SBRT-treated group achieved a significantly improved mPFS (20.2 vs.12.5 months, *p* < 0.001) and median OS (25.5 vs. 17.4 months, *p* < 0.001).

The oligometastatic state is considered independent of the type of the primary tumor [22]. However, its indiscriminate adoption may be inappropriate considering the heterogeneity of various OMDs. For example, contrary to the positive results of the SINDAS trial that examined the effect of MDT consolidation in the setting of EGFR-mutated NSCLC, the NRG-BR002 trial, which tested the effect of local consolidation in oligometastatic breast cancer patients, found no significant difference in PFS or OS [23].

Omission of systemic therapy should be considered with caution in highly selected patients, as solely treating the overt metastatic lesions could compromise outcomes in certain malignancies. SABR-COMET compared systemic SOC therapy with or without the addition of SBRT [4]. SINDAS enrolled patients with EGFR-mutated NSCLC who responded to systemic therapy and then received local consolidation [24]. The accruing phase III SABR-COMET 3 and 10 studies, would further assess the impact of SBRT in addition to systemic therapy in patients with up to 3 or 10 metastatic lesions, respectively [2, 3]. Conversely, STOMP and ORIOLE excluded patients who initiated androgen deprivation therapy.

Aggressive local therapy with SBRT is associated with durable disease control owing to low rates of local failure [18]. However, the treatment carries a risk for serious side effects. A meta-analysis of prospective trials evaluating SBRT in oligo-metastatic patients demonstrated acute grade 3–5 toxicity rates between 0% and 20% and late grade 3–5 toxicity between 0% and 10% [6]. While it is perceived to be potentially curative in patients with a limited number of metastatic lesions, long-term data is lacking. Hence, the current therapeutic goal should be prolonged OS and improved QoL. It is imperative to highlight treatment objectives to patients and confirm compatibility with their wishes. In cases where long-term PFS or potential cure is pursued, patients may be willing to accept a risk for severe toxicity that could be associated with long-term detrimental effects on their QoL. However, when the objective is palliative, any intervention must be meticulously scrutinized.

Compared with the limitations of the HR and the potential misleading interpretation of the median, while less familiar, the RMST difference is a robust and intuitive measure of outcomes. It can be explained as the average time benefit from the proposed treatment subject to the follow-up period. Some trials, such as SINDAS, yielded comparable estimates, with RMST and median OS calculated at 7.9 and 7.4 months, respectively. Nonetheless, while the median indicates the chances of surviving 7.4 months are as likely as not, it does not provide information about the expected survival time which is provided by RMST with 7.9 months. Further, RMST is more stable and precise due to narrower confidence interval than that of the median [10]. For other trials, the median and RMST estimates differed substantially without any significant correlation to the HR. Notably, the RMST for PFS analysis of SABR-COMET and bRFS analysis of STOMP suggested a median benefit that is underestimated by more than two-fold, with absolute difference of 5.7 and 4.3 months, respectively. In contrast, the RMST for OS analysis of SABR-COMET and Gomez et al. implied an overestimated median survival benefit with an absolute difference of 9.7 and 5.5 months, respectively. Furthermore, the difference in mPFS was not estimable in ORIOLE as the median for the SBRT group was not reached, which restricts the clinical interpretability of the outcome. Contrarily, the RMST difference was estimated to be 6.2 months. Unlike the HR or median, the RMST consistently results in clinically interpretable summaries of the treatment effect.

RMST has been gaining recognition for its utility in survival analysis and is increasingly employed for reporting study outcomes within the literature [25–27], major oncology conferences [28, 29], and support the approval of drugs by the FDA [30–32]. Serving as either a complimentary metric or alternative to the HR, RMST can be used to describe clinical endpoints such as OS and PFS. Notably, when RMST is reported it is often used when the PH assumption is not met.

Although the conventional measures HR and median have their benefits, RMST can serve as a robust alternative for both metrics, even when the PH assumption holds. The distinct advantages of the RMST lie in its capacity to provide a single, precise, model-free, clinically interpretable, time-scale summary of survival. However, no single summary measure may always adequately characterize the results. Given that clinical trialists customarily report HR and median, we fill it is instructive to compare these with RMST, even when the PH assumption is satisfied. Conversely, when the PH assumption is violated, RSMT should substitute HR as a summary measure and prespecified in the study protocol.

### Limitations

The limitations of this study include the reliance on small, mostly phase II trials, which may not be thoroughly applicable to larger populations. Additionally, there is a high degree of variation between the studies in terms of patient characteristics, prognosis of the primary disease, endpoints, and follow-up time. The former can make interpretation of the results challenging. Although our main goal was to analyze local treatment of OMD with RT, some studies included both surgery and RT, leading to potential selection bias between these two modalities. Notably, surgery remains the main modality for some indications, such as oligometastatic liver disease in colorectal cancer [33]. Furthermore, only three studies reported on OS, providing a limited view of the gold standard, most significant endpoint in establishing clinical benefit. Additionally, the use of RMST as a measure of treatment efficacy, similarly to HR, is follow-up time-dependent and may lead to over- or underestimation of the effect. Furthermore, any survival analysis is based on the assumption of uninformative censoring, which can affect the validity of the results [34–36]. In addition, the study did not evaluate the statistical robustness of the results [37].

## Conclusion

The RMST method is a robust alternative to the HR in summarizing survival data and may be more effective in communicating treatment effects to patients and healthcare professionals. In this report, it supports the potential benefit of MDT in patients with oligometastatic solid malignancies.

### Supplementary information

Supplementary material
