# Supplementary material for: Assessing the effect of metastasis-directed therapy in oligometastatic disease using the restricted mean survival time
Source: Br J Cancer. 2024 May 6;130(12):1929–35. doi: 10.1038/s41416-024-02700-z (PMC11183092; doi:10.1038/s41416-024-02700-z)
Supplement: Supplementary file 1 — Supplementary material [file 41416_2024_2700_MOESM1_ESM.docx]

**Supplementary material**


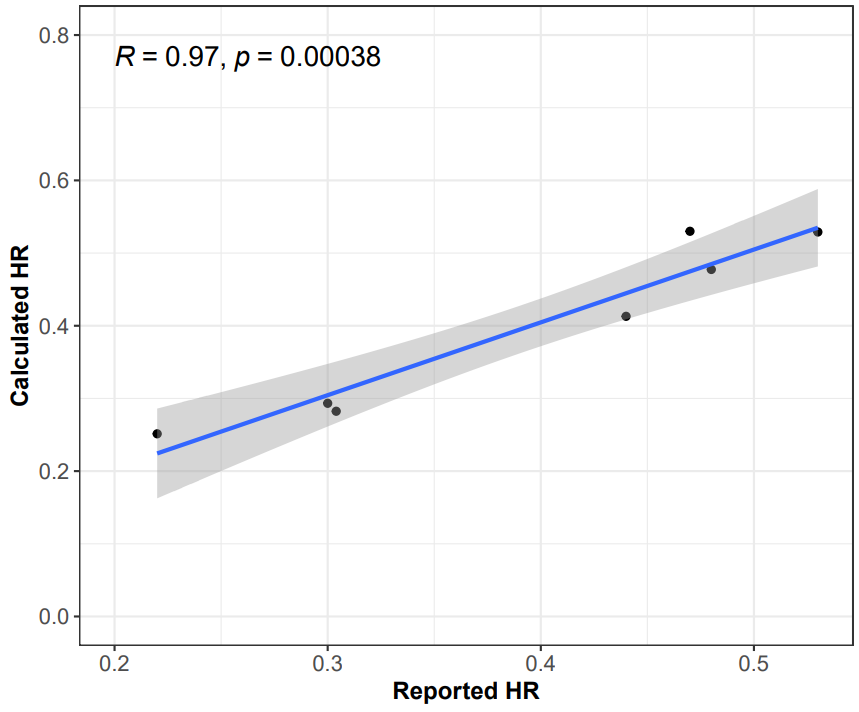


Supplementary Figure 1. Relationship between the reported and calculated hazard ratio (HR). Shown is a significant correlation between the HR, as reported in the publications, and the calculated HR for the surrogate endpoints and overall survival. Correlation was calculated using Pearson’s correlation coefficient.
